# Lung Nodule Detection in CT Images Using Statistical and Shape-Based Features

**DOI:** 10.3390/jimaging6020006

**Published:** 2020-02-24

**Authors:** Noor Khehrah, Muhammad Shahid Farid, Saira Bilal, Muhammad Hassan Khan

**Affiliations:** 1 Punjab University College of Information Technology, University of the Punjab, Lahore-54000, Pakistan; noor.khehrah@pucit.edu.pk (N.K.);; 2 Department of Radiology, General Hospital, Lahore-54000, Pakistan

**Keywords:** computer-aided detection (CAD), nodule detection, lung cancer, computed tomography (CT)

## Abstract

The lung tumor is among the most detrimental kinds of malignancy. It has a high occurrence rate and a high death rate, as it is frequently diagnosed at the later stages. Computed Tomography (CT) scans are broadly used to distinguish the disease; computer aided systems are being created to analyze the ailment at prior stages productively. In this paper, we present a fully automatic framework for nodule detection from CT images of lungs. A histogram of the grayscale CT image is computed to automatically isolate the lung locale from the foundation. The results are refined using morphological operators. The internal structures are then extracted from the parenchyma. A threshold-based technique is proposed to separate the candidate nodules from other structures, e.g., bronchioles and blood vessels. Different statistical and shape-based features are extracted for these nodule candidates to form nodule feature vectors which are classified using support vector machines. The proposed method is evaluated on a large lungs CT dataset collected from the Lung Image Database Consortium (LIDC). The proposed method achieved excellent results compared to similar existing methods; it achieves a sensitivity rate of 93.75%, which demonstrates its effectiveness.

## 1. Introduction

Lung cancer has a high causality rate. According to a survey [1], more than 1.37 million people died from Lung cancer throughout the world only in 2008. The American Cancer Society has projected 1.74 million new cancer cases and 0.61 million cancer deaths in the year 2018 [2]. Two main reasons for the high mortality rate in lung cancer are the delay in early diagnosis and the poor prognosis [3]. The study reveals that 70% of lung cancers are diagnosed in too advanced stages, where the cancer prognosis is ineffective. Therefore, early diagnosis of cancer is momentous for increasing the patient’s chances of survival.

Computed Tomography (CT) scan images are utilized for cancer diagnosis; the radiologists examine them to detect and classify the nodules into malign and benign [4]. However, these methods require highly skilled radiologists who are not available, particularly, to the people of remote and poor regions. Moreover, there is a high risk of human error in manual examination, and thus Computer Aided Detection (CAD) systems are needed that can help the radiologists in the diagnosis and help decrease the rate of false reports. Digital image processing techniques can be used to detect the nodules, their type, size, and other features from CT scans.

Medical image processing has been extensively and increasingly applied to design expert support systems for the diagnosis of numerous diseases, e.g., arthritis detection [5,6], parasite detection [7,8,9], lung cancer detection [10,11,12], and rehabilitation [13,14,15]. A significant amount of research is being done in the early lung cancer diagnosis using CAD systems [16,17,18,19,20]. The need for automated systems arises because of the nature of the data—CT scans—used in lung cancer diagnosis. A lung CT scan contains usually more than 250 images per scan. Examining this extensive dataset for each patient is quite a challenging, time-consuming, and a tedious task for a radiologist. Moreover, the nature of nodules which decides the fate of a patient is also very complicated, as their shape and size varies from slice to slice. Sometimes they are attached to other pulmonary structures, such as vessels or bronchioles. The color in which they appear on CT scans may also differ. These factors add to the complexity of identifying them. However, the same elements, once recognized, help researchers in defining the course of their methodology.

A fundamental step in lung nodules detection is the accurate lung segmentation from the CT image. For this purpose, numerous techniques have been proposed for efficient lung segmentation. Some algorithms require a few seed pixels on the lungs region in the image and then utilize region-growing techniques to segment the lungs e.g., [21,22,23,24]. The algorithm in [21] extracts the chest out of complete CT images using a region-growing algorithm taking four seed points as input. The same technique was used to segment lungs from the chest. Cascio et al. [23] also use a region-growing algorithm for lung segmentation. They use the center pixel of the slice as seed. The method in [22] segments the nodules with the assistance of the radiologists. The technique proposed in [25] divides the chest CT scan into four classes: lung wall, parenchyma, bronchioles, and nodules. The active contour technique is used to segment lungs from the CT images.

Numerous methods in the literature use nodule intensity or color thresholding to detect nodules in CT images, e.g., [26,27,28,29,30,31,32]. Clifford et al. [26] preprocessed the CT image with bi-histogram equalization to improve its sharpness. The resultant image is thresholded to obtain connected components. The area and pixel values of these segmented regions are passed as features to fuzzy inference system for nodule detection. Another rule-based technique for lung segmentation is proposed in [27]. A nodule size is used in [28] as the standard to detect potential nodule regions. Local maxima are found for each subvolume with values and size larger than the standard nodule in 3D space. Messay et al. [29] use thresholding for initial segmentation, and then a rule-based analysis on the anatomical characteristics of bronchioles present between lungs. It detects and segments nodule candidates simultaneously, unlike the methods discussed previously. The algorithms in [30,31] are also thresholding-based methods for lung nodule classification.

Template matching has also been explored for nodule detection, e.g., [33,34,35]. In such methods, the template nodule images are searched in the target image in order to find the nodules. Ozekes et al. [33] developed a 3*D* template for the detection of nodule candidates. The template is convoluted to the Region Of Interests (ROIs) and extracted by applying an eight-directional search on the lungs region. In [36], thresholding and binarization of the CT image are used for lung segmentation. A multi-scale filtering is used for the detection of nodules.

Nodules have been shown to possess certain properties, such as shape, color, and intensity, which have been used to design a composite discriminative feature. The features are then classified to detect the nodule through different classification methods. The methods in [37,38,39,40,41] are a few such examples. The algorithm proposed in [37] extracts six features from each slice and forms feature vectors, which are classified using a support vector machine to identify the nodules. A two-dimensional (2D) multiscale filter is proposed in [39] for the detection of lung nodule candidates. The shape features for each segmented region are used to reduce the false positive rate through a classifier. The method proposed in [41] uses surface triangulation on different threshold values for the detection of nodule candidates and neural network classifiers are used to separate the nodules from the non-nodules. Deep learning techniques have also been explored lately for lung nodule detection, e.g., [42,43,44]. The research in [45,46,47] presents the recent developments in automatic lung nodule detection.

In this paper, we propose a CAD system that automatically extracts the lungs from chest CT scans and processes these segments to detect nodules. The major contributions of this paper are as follows:

- In most existing CAD-based nodule detectors, the lungs are manually marked by the radiologist, which is a tedious and time-consuming task. In the proposed algorithm, the lungs are automatically segmented from the CT images without any user intervention;
- Nodules can have different regular and irregular shapes and sizes. Some existing techniques use a few shape templates to detect the nodules, however the proposed algorithm is independent of the nodule shape and size;
- The proposed system uses basic image processing techniques, e.g., histogram processing, morphological operators, connected components analysis, etc., which makes it implementable on simple computers, making it an efficient and cost effective solution;
- In an experimental evaluation carried out on a standard LIDC dataset, the proposed system achieved high sensitivity and accuracy, outperforming the existing similar techniques.

## 2. Materials and Methods

The strategy proposed for lung nodule detection comprises two major phases. In the first phase, lung are segmented from the Digital Imaging and Communications in Medicine (DICOM) CT scans, and in the second phase nodules are detected from the lungs. In most existing techniques, the lungs segmentation is performed in semi-automatic ways, where the radiologist assists the system by either specifying the region of interest or by drawing a few scribbles on the target object. Subsequently, based on the user input, the lungs are extracted from the rest of the image. In the proposed framework, the lung segmentation is totally automatic and no assistance from the user is needed. The proposed segmentation technique constructs a histogram of the given image and analyzes it to automatically select a threshold. Based on this threshold the outer region in the image is identified and dropped, and the rest of the region is further processed to extract the lungs. This process involves morphological operations and connected components analysis.

**Figure 1:**
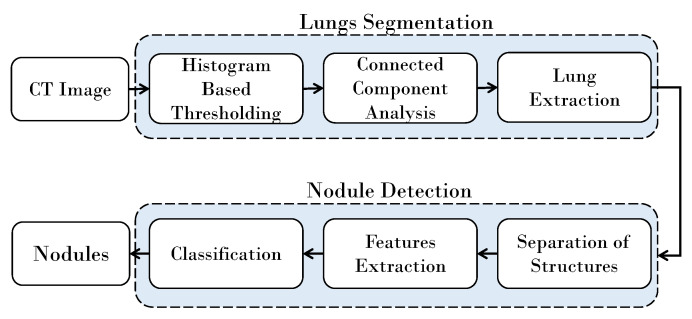
Block diagram of the proposed algorithm.

In the second phase, the nodules are detected from the segmented lungs. It is achieved by separating the inner structures of nodules, bronchi, and blood vessels from the parenchyma region. A model is trained using the statistical and shape-based features of the nodules, and a support vector machine-based classifier is used to separate the nodules from the bronchi and the blood vessels. A block diagram of the proposed algorithm is shown in Figure 1.

### 2.1. Lung Segmentation

The medical images are not in the conventional image formats such as PNG or JPEG, as they are conducted under a specified constrained environment, which has a direct impact on the image attained. *Digital Imaging and Communications in Medicine* (DICOM) is a standard for storing and transmitting digital images, enabling the integration of different medical imaging devices, e.g., scanners, servers, workstations, and printers. A DICOM image may also contain the information of the patient, date, and many other data that are not required in lung segmentation. To conveniently perform the image processing tasks, we convert the DICOM images to the loss-less Portable Network Graphics (PNG) format. When a DICOM image is converted to PNG format, the personal information of the patient and all tags which come with a DICOM format get removed so as to preserve the individual’s privacy.

**Figure 2:**
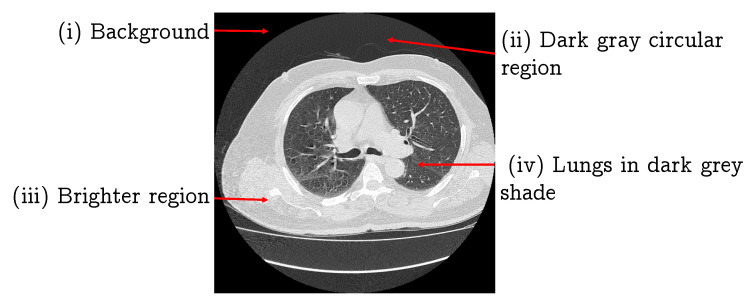
A CT image of lung and proposed division into four regions.

The lung segmentation is considered a fundamental activity in nodule CAD systems, as the performance of the later stages in such an analysis largely depends on the segmentation accuracy. In this section, we propose a lung segmentation algorithm that utilizes the histogram and morphological image processing techniques. The converted PNG image has four components: (i) a black background; (ii) a dark gray circular region; (iii) a brighter region; and (iv) the lungs in a dark gray shade, as shown in Figure 2. Our region of interest is the lungs, thus here we remove the first two components.

**Figure 3:**
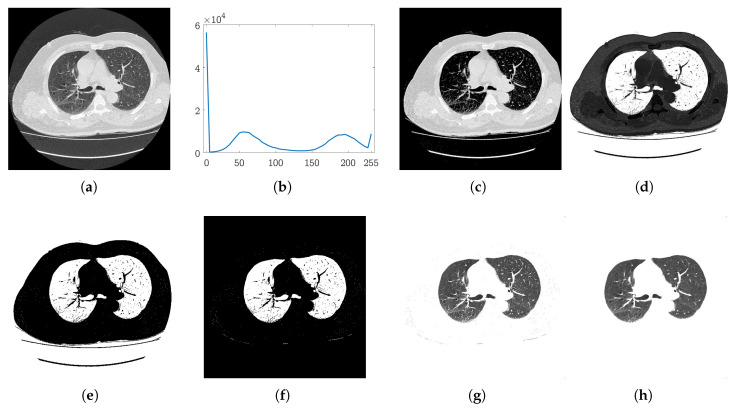
Automatic histogram based on initial segmentation: (**a**) Input CT slice; (**b**) histogram of the image in (a); (**c**) the results after thresholding the image with the threshold estimated from the histogram in (b); (**d**) the complemented image of (c); (**e**) binary segmentation map; (**f**) map after connected components-based refinement; (**g**) detected lungs; (**h**) lungs after noise removal.

To reduce the image to our ROI, we perform thresholding. Thresholding is mainly dependent on the value of the threshold, and this value is usually user specified. In our case, we compute the threshold for each slice from the histogram of the input image. Figure 3b shows the histogram of a sample lung image shown in Figure 3a. It can be noted that the histogram has four prominent peaks. One very high peak is at 0, which corresponds to the black background in the image. The second peak around the gray level 60 is formed from the dark gray circular region covering the bright region. These two peaks correspond to the regions (i) and (ii) as discussed above. A high peak at 255 corresponds to the white region mainly around the lungs, and the third peak around gray level of 210 in this example is formed by the intensities of the lungs and patches inside the bright region around the lungs. Thus, by dropping the pixels that fall in the first two peaks, we can remove the background regions (i) and (ii). The valley where the second peak ends serves as a separator between regions i–ii and regions iii–iv. This valley can be estimated by computing the second minima of the histogram. The value of the second minima is used as a threshold to remove the regions (i) and (ii) from the CT scan. Figure 3c shows the result achieved after thresholding the image (Figure 3a) with the estimated threshold.

Let *I* be an input lung image of size M×N after pre-processing. A histogram *H* of *I* is computed using the step size κ, and the 2nd local minima λ of *H* is computed and used in thresholding the image *I* to remove the background from the image.

(1) $$\hat{I}\left(x,y\right)=\left\{\begin{array}{cc} I(x,y) & \mathrm{if}\;I(x,y)>\lambda \;,\\ 0 & \mathrm{otherwise} \end{array}\right.$$

where $\hat{I}$ is the thresholded image. Conventionally, the white color is used to represent the foreground and the black color is used for the background; we then complement the resultant image. Figure 3d shows the complemented image $\bar{I}$ obtained from $\hat{I}$. The next step is to separate the lung region from its surrounding bright region. The histogram-based thresholding applied to remove the background regions is not effective in this case, as the bright region covering the lungs contains patches of significantly different intensities (Figure 3c). For this purpose, we use the Otsu method [48] to separate the lungs from the surrounding region. Let τ be the threshold obtained from [48], which is used to obtain the binary mask *B* of lungs:

(2) $$B\left(x,y\right)=\left\{\begin{array}{cc} 1 & \mathrm{if}\;\bar{I}\left(x,y\right)>\tau ,\\ 0 & \mathrm{otherwise} \end{array}\right.$$

The resultant binary mask is shown in Figure 3e. It can be observed that the mask still contains a few unwanted objects. If we see this image as components of pixels, it can be concluded that our region of interest is the two separate components enclosed in the largest component. To this end, we compute the connected components [49] of the mask to achieve our goal of getting a binary map of the lungs.

We simply determine the largest component that corresponds to the region enclosing the lungs, as evident from Figure 3e. Hence after determining this component, we shred it off to obtain the lungs mask *M*. Moreover, a morphological dilation operation is also applied to the extracted map to include the false negatives inside the lungs. The resultant map is shown in Figure 3f. Using this map, we segment the lungs out of the original image *I*, shown in Figure 3g. Salt and pepper noise can be noted in the mask (Figure 3f) and in the final segmented lungs (Figure 3g). We apply a median filter of size 3×3 to remove this noise. The final segmented lungs are shown in Figure 3h.

### 2.2. Nodules Detection

In this phase, first the inner structures of the lungs, i.e., nodules, bronchi, and blood vessels, are separated from the parenchyma region. The inner structures in the lungs appear as bright spots (Figure 3h), which can be easily separated through thresholding as the intensity levels of the parenchyma region and inner structures are distinguishably different. The Otsu method [48] is used on the segmented lungs to separate the inner structure vessels, bronchi, and nodules (if there are any) from the rest of the region.

**Figure 4:**
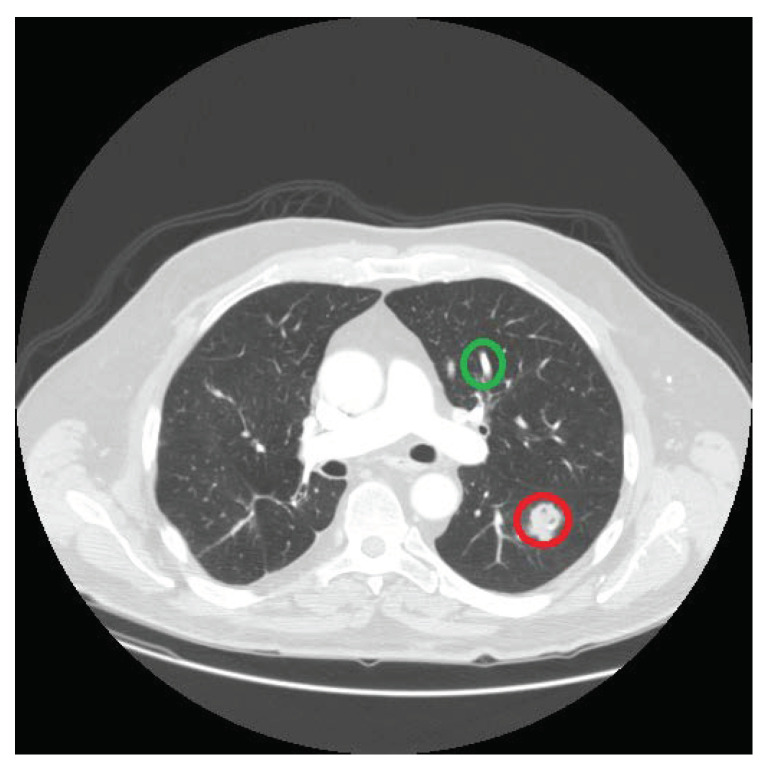
The region circled in red is a nodule and the region circled in green is a non-nodule.

The nodules differ from other structures present in lungs in many aspects. One key difference is their shape, and we exploit this property to isolate the nodules from the non-nodules structures, i.e., vessels and bronchi. The nodules are spherical in shape, whereas the vessels and the bronchioles are cylindrical, as shown in Figure 4. We use the size invariant round/near-round shape detection algorithm proposed in [50] to identify the circular shapes in the detected set of structures. It returns the centers of the potential nodule locations, which are used as seed points in a region-growing algorithm [51] to extract the nodule candidates. In contrast to existing nodule template-based techniques, the proposed strategy enables us to extract nodules of any shape, making our methodology independent of any nodule template.

Let $\left\{c_{1},c_{2},c_{3},\cdots ,c_{n}\right\}$ be the centers of the shapes extracted using algorithm [50]. The centers are passed to the region-growing algorithm as seeds, which returns us the corresponding *n* nodule candidate regions $\left\{A_{1},A_{2},A_{3},\cdots ,A_{n}\right\}$. We compute different features of nodules and construct a feature vector to discriminate nodules from the other inner structures. We exploit different statistical properties, shape-based features, and across-slice characteristics of the candidate regions to design a discriminative feature vector. In particular we use the following statistical and shape properties of candidates regions:

1. Mean ($\mu_{i}$) represents the average value of the region $A_{i}$:
  (3) $$\mu_{i}=\frac{1}{n}\sum_{x\in A_{i}}x;$$
2. Median ($me_{i}$) is the mid-point of $A_{i}$ when arranged in non-decreasing order;
3. Mode ($mo_{i}$) is the most repetitive element of the data in $A_{i}$;
4. Variance ($\sigma^{2}$) represents to what extent the data varies from the mean value. For region $A_{i}$, $\sigma_{i}^{2}$ is:
  (4) $$\sigma_{i}^{2}=\frac{1}{\left|A_{i}\right|}\sum_{x\in A_{i}}{\left(x-\mu_{i}\right)}^{2}$$
  where $\left|A_{i}\right|$ and $\mu_{i}$ represent the size and mean of the region $A_{i}$, respectively;
5. Standard deviation σ is the square root of variance:
  (5) $$\sigma_{i}=\sqrt{\sigma_{i}^{2}};$$
6. Consistency feature: one more important feature is based on the shape of the lesion and its appearance in the colocated slices of the CT scan. That is, if a nodule exists in one slice, it must also appear in the preceding slices or in the succeeding slices of a CT scan. On the other hand, the vessels and bronchi transform further into new shapes, so if they are detected in one slice there are high chances that they will not be present in the exact location in the next slice of the series. This property is an important feature of nodules. Therefore, the center points detected in slice $S_{j}$ are traced in a window of size 2k+1 in adjacent slices. That is, the center points detected in slice $S_{i}$ will be compared with the center points identified in *k* previous and *k* next slices,
  $$\underset{\mathrm{Slice}\;\mathrm{Search}\;\mathrm{Window}}{\underset{︸}{S_{j-k},\cdots ,S_{j-2},S_{j-1},S_{j},S_{j+1},\cdots ,S_{j+k}}}$$
  and we assign a center point 1 if it exists in any of those 2k slices, and 0 if it does not exist in any of them. This consistency feature for candidate $A_{i}$ in the current slice $S_{j}$ is represented as $t_{i}$. In this paper, we use a window of size 3, (k=1), to determine the the value of feature $t_{i}$ for each location $A_{i}$ in a given slice.

**Figure 5:**
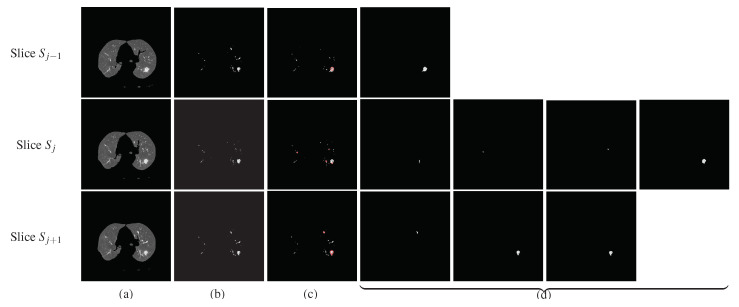
Spherical shape detection (reader is requested to magnify the images to see the details). The 2nd row shows the processing of the slice under consideration, whereas the 1st and the 3rd rows show the processing of previous and next slices, respectively. (**a**) Segmented lungs, (**b**) the extracted internal structures, (**c**) the detected circle in red boundaries, and (**d**) the final detected nodules.

Figure 5 shows an example of computing the consistency feature for a sample slide $S_{j}$. The preceding slide is $S_{j-1}$ and the next slide is $S_{j+1}$. The three slices are shown in column (a), the lungs detected using the proposed algorithm are shown in (b), and the inner structures identified by the proposed technique are shown in (c). The centers of the round/near-round regions detected by our method are highlighted using small red circles in (c). These centers are passed to the region-growing algorithm to mark the shape of the objects. The spherical objects detected in each slice are shown in (d) separately. One potential nodule is detected in slice $S_{j-1}$, four candidates are detected in slice $S_{j}$, and three candidates are detected in slice $S_{j+1}$, as is shown in column (d) of Figure 5. It can be noted that if only one candidate of slice $S_{j}$ is found in the previous and also in the next slices of $S_{j}$, it is present in the last figure (from left-to-right) of each row. This object of slice $S_{j}$ is taken as a nodule candidate, the other three regions which could not be traced either in $S_{j-1}$ or in $S_{j+1}$ are marked as vessels or bronchi and are dropped from further processing.

All the features described above are computed for each nodule candidate region $A_{i}$ in a slice $S_{j}$ and then combined to obtain a feature vector $F_{i}$,

(6) $$F_{i}=\left[\mu_{i},me_{i},mo_{i},\sigma_{i}^{2},\sigma_{i},t_{i}\right].$$

Based on feature *F*, the selected regions are classified as nodules and non-nodules using a Support Vector Machine (SVM). Training is done by using features of a training dataset, and then these same features are calculated for the testing dataset and passed to the model for classification. We used the LIBLINEAR SVM library [52] in our implementation for SVM classification.

## 3. Experiments and Results

**Figure 6:**
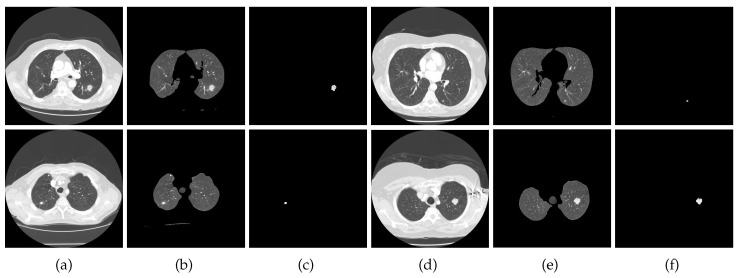
Nodule detection results: (**a**,**d**) input CT slice, (**b**,**e**) segmented lungs, (**c**,**f**) detected nodule.

To assess the performance of the proposed algorithm, we performed a large set of experiments on a standard lung CT dataset. The performance is objectively computed and the results are also compared with 10 existing similar techniques, including [11,21,29,30,31,36,53,54,55,56]. In the region-growing algorithm, the maximum intensity distance was set to 0.18. In all experiments, κ was set to 5 and the window of size 3 was used in the computation of the nodule consistency feature vector. Figure 6 presents a few more results of nodule detection achieved by our method on images from the test dataset.

### 3.1. Evaluation Dataset

The dataset of lungs CT scans was collected from the Lung Image Database Consortium (LIDC) database [57]. This collection contained 70 cases of Lung scan acquired using different CT scanners. Four radiologists tagged these scans and the tagging was done in two phases. In the first phase, each radiologist tagged the scans independently, and in next phase, results from all radiologists were compiled together and then given to each radiologist for a second review. In this phase, all radiologist were able to review their previous annotations as well as annotations done by the other radiologists. Each of these 70 cases is a series of 250–350 images.

### 3.2. Performance Evaluation

In this section, we evaluate the performance of the proposed lung nodule detection algorithm using different statistical metrics. There are four possible outcomes of the proposed algorithm when run on a test image: true positive (TP), true negative (TN), false positive (FP), and false negative (FN). True positives (TP) means that nodules exist in the image and they are detected correctly, and true negative (TN) means that there are no nodules in the images and this is correctly identified. False positive (FP) occurs when no nodule exists in the image but is incorrectly detected by the algorithm, and false negative (FN) occurs when a nodule is missed by the algorithm.

To objectively quantify the performance of the proposed algorithm, we chose various statistical measurement parameters: sensitivity, specificity, precision, accuracy, and F score. Sensitivity, also called Recall, is a measurement of true positive rate, i.e., a nodule is tagged as the nodule:

(7) $$Sensitivity\;\mathrm{or}\;Recall=\frac{TP}{TP+FN}.$$

Specificity measures the true negative rate, i.e., non-nodule is tagged as non-nodule. It is computed as:

(8) $$Specificity=\frac{TN}{TN+FP}.$$

Precision demonstrates how much is the algorithm precise in detecting true positive results:

(9) $$Precision=\frac{TP}{TP+FP}.$$

Accuracy is the measurement of how well the binary classifier correctly identifies or excludes a condition:

(10) $$Accuracy=\frac{TP+TN}{TP+TN+FP+FN}.$$

When the positive and negative classes in the binary classification are highly unbalanced, the accuracy and precision metrics can be delusive [58], and F measure is considered to be more reliable in such situations. The F measure is a weighted harmonic average of the precision and recall. Therefore, this score takes both false positives and false negatives into account and provides the overall accuracy of the model. The $F_{\beta }$ score is used in our analysis. $F_{\beta }$ measures the effectiveness of retrieval with respect to a user that attaches β times as much importance to recall as precision [59,60]:

(11) $$F_{\beta }=\left(1+\beta^{2}\right)\cdot \frac{\mathrm{precision}\cdot \mathrm{recall}}{\beta^{2}\cdot \mathrm{precision}+\mathrm{recall}}$$

We chose the widely used $F_{0.5}$ in our evaluation.

To further investigate the performance of the proposed method, we also performed a Matthews Correlation Coefficient (MCC) [61] test. It measures the quality of the binary classification and is considered to be more truthful and informative than other parametric statistical measures [62]. The value of MCC varies between −1 and +1, where the maximum value +1 represents a perfect prediction, −1 indicates total disagreement between prediction and ground truth, and 0 represents that it is no better than random prediction. It is computed as:

$$\mathrm{MCC}=\frac{\left(TP\times TN)-(FP\times FN\right)}{\sqrt{\left(TP+FP)(TP+FN)(TN+FP)(TN+FN\right)}}$$

This dataset was divided into two subsets, one used for training and other used for testing. We used different divisions—40:60, 50:50, 60:40, 70:30—for training and testing, respectively, to analyze the performance of the proposed algorithm. The results are reported in Table 1. The statistics show that the best results were gained at the 70:30 percent division. The same division trend was observed in [21], therefore in our experiments we used 70% of the dataset for training and 30% for testing. In our experiments, we performed a 4-fold cross-validation. The statistics presented in Table 1 show that our algorithm achieves an accuracy of 0.92, F score of 1.0976, and MCC value 0.8385. The results reveal that the proposed method is reliable for lung nodule detection.

### 3.3. Performance Comparison

We also compared the results of our method with ten existing similar nodule detection algorithms. The list of compared methods includes [11,21,29,30,31,36,53,54,55,56]. Although it is difficult to compare their performance because it depends on the image datasets and detection parameters, it is still important to attempt making a relative comparison. The Sensitivity measure is widely used to report such results, as can be found in [11,21,29,30,31,36,53,54,55,56], therefore we also used a sensitivity metric to present the comparison. The results of the comparison are presented in Table 2. To further analyze the performance of the proposed and compared methods, we also report the average FPI (False Positive per Image) and average FPE (False Positive per Exam) metric values. The FPI is the ratio of falsely accepted negative samples to the total number of images, that is,

(12) $$FPI=\frac{\mathrm{False}\;\mathrm{Positives}\;\left(\mathrm{FN}\right)}{\mathrm{Total}\;\mathrm{number}\;\mathrm{of}\;\mathrm{tested}\;\mathrm{images}}$$

Similarly, the false positive per exam (FPE) is the ratio of false positives to the total number of cases evaluated in the experiment.

The statistics presented in Table 2 reveal that our method achieved convincing results. In the sensitivity measure, our method achieved 93.75% sensitivity, outperforming all compared methods. In terms of FPI and FPE, our method achieved 0.13 and 0.22 scores, respectively, and the best results were achieved by Stelmo [21]. However, the results were computed only for 29 scans compared to our dataset of 75 scans. Moreover, our method has a better sensitivity rate than [21]. From the results of the objective performance evaluation, one can conclude that the proposed method is effective and accurate for lung nodule detection. Moreover, in contrast to most compared methods which are semi-automatic, our method is fully automatic. All the thresholds and other parameters used in our method are automatically estimated, and no external assistance is needed at any stage of the algorithm. These characteristics make the proposed algorithm ideal for lung nodule detection.

### 3.4. Computational Complexity Analysis

The proposed algorithm is implemented in Matlab and is made freely available for peer and public use on the project web-page (http://www.di.unito.it/~farid/Research/hls.html). We performed an execution time analysis of the proposed and the compared methods. To this end, the proposed algorithm was executed on the test dataset and average execution time was computed. The experiment was executed on an Intel® Core™ i5 processor with 4GB RAM and a 64-bit operating system. Our method takes approximately 12 s to detect nodules from a slice, which is quite efficient considering that a nodule feature is computed temporally by locating the nodule candidates in the current slice and tracing them in adjacent images. By contrast, the Stelmo [23] and Froz [53] methods take on average 90 s and 9.2 s, respectively, to examine a CT image. An efficient implementation of the proposed algorithm can further reduce its running time.

## 4. Summary and Conclusions

In this paper, we presented an automated system for the detection of lung nodules from CT images. The functionality of our system can be divided into two phases: first, the lung segmentation from the chest CT scan, and second, the nodule detection. The lung segmentation in the proposed algorithm is performed automatically, a novel histogram-based threshold estimation technique is proposed in this regard. This method shows that the classification of structures is based on their dimensionalities. A large number of ROIs extracted from lungs after the lung segmentation phase is a challenge for accurate classification. This problem is addressed by using the shape feature and the property that a nodule exists in consecutive slices. The testing stage of the SVM classifier resulted in 0.13 false positives per slice. The proposed algorithm achieved excellent results with a sensitivity of 0.9375, accuracy of 0.92, and a Matthews correlation coefficient of 0.8385. These results and the comparison with the existing CAD systems reveal the effectiveness of the proposed method.

The high incident rate of lung cancer and the late diagnosis show that this automated system can be conducive in the early scan stages. An exam of lung CT scan consists of a long series of images, and this system can analyze these images fast and reduce the risk of human error. This system can be used as the first step of a diagnosis; the marked cases can be passed for medical analysis for further studies and confirmation. We need to mention here that public hospitals lack in number of specialists and the number of patients visiting the hospital is enormous as compared to the number of doctors available. The proposed method can be used to lower this burden. The method can be implemented for preliminary scans and a radiologist can validate these results. Thus this system will be an assistant to the radiologists. This system is economical to design as it requires regular computers for deployment, which are usually already available in hospitals and clinics or can be easily procured.

## Figures and Tables

**Table 1:** Performance evaluation of the proposed algorithm on different training and testing dataset divisions. The division shows the ratio of training to test dataset size. The best results are marked in bold.

Division	Sensitivity	Specificity	Precision	Accuracy	F Score	MCC
40:60	0.8611	0.8824	0.8378	0.8736	1.0276	0.3491
50:50	0.8621	0.8864	0.8333	0.8767	1.0274	0.3908
60:40	0.9200	0.8889	**0.8519**	0.9016	1.0866	0.5838
70:30	**0.9375**	**0.9118**	0.8333	**0.9200**	**1.0976**	**0.8385**

**Table 2:** Performance evaluation of the proposed algorithm and the compared methods. Size represents the number of scans in the dataset. FPI: False Positive per Image; FPE: False Positive per Exam.

Method	Year	Database	Size	Sensitivity	FPI	FPE
Dolejsi [36]	2009	TIME-LIDC-ANODE	38	89.60	12.03	-
Golosio [31]	2009	LIDC	484	71.00	-	4
Messay [29]	2010	LIDC	84	82.66	-	3
Tan [30]	2011	LIDC	399	87.50	-	4
Stelmo [21]	2012	LIDC	29	85.93	0.001	0.14
Teramoto [54]	2013	LIDC	84	80.00	-	4.2
Bergtholdt [56]	2016	LIDC-IDRI	243	85.90	-	2.5
Wu [55]	2017	LIDC-IDRI	60	79.23	-	-
Froz [53]	2017	LIDC-IDRI	833	91.86	-	-
Saien [11]	2018	LIDC/LIDC-IDRI	70	83.98	0.02	-
Ours	2019	LIDC	75	93.75	0.13	0.22
